# Publisher Correction: Long-haul optical transmission link using low-noise phase-sensitive amplifiers

**DOI:** 10.1038/s41467-018-05591-w

**Published:** 2018-07-31

**Authors:** Samuel L. I. Olsson, Henrik Eliasson, Egon Astra, Magnus Karlsson, Peter A. Andrekson

**Affiliations:** 1 Department of Microtechnology and Nanoscience, Chalmers University of Technology, SE-412 96 Gothenburg, Sweden; 2 Thomas Johann Seebeck Department of Electronics, Tallinn University of Technology, 19086 Tallinn, Estonia; 3 Nokia Bell Labs, 791 Holmdel Road, Holmdel, NJ 07733 USA

Correction to: *Nature Communications*; 10.1038/s41467-018-04956-5; published online: 28 June 2018

The original version of this Article incorrectly listed an affiliation of Samuel L.I. Olsson as ‘Thomas Johann Seebeck Department of Electronics, Tallinn University of Technology, Tallinn 19086, Estonia’, instead of the correct ‘Present address: Nokia Bell Labs, 791 Holmdel Road, Holmdel, NJ 07733, USA’. Similarly, Egon Astra had an incorrect affiliation of ‘Present address: Nokia Bell Labs, 791 Holmdel Road, Holmdel, NJ 07733, USA’, instead of the correct ‘Thomas Johann Seebeck Department of Electronics, Tallinn University of Technology, Tallinn 19086, Estonia’. This has been corrected in both the PDF and HTML versions of the Article.

